# Situational awareness, relational coordination and integrated care delivery to hospitalized elderly in The Netherlands: a comparison between hospitals

**DOI:** 10.1186/1471-2318-14-3

**Published:** 2014-01-10

**Authors:** Jacqueline M Hartgerink, Jane M Cramm, Annemarie JBM de Vos, Ton JEM Bakker, Ewout W Steyerberg, Johan P Mackenbach, Anna P Nieboer

**Affiliations:** 1 Department of Social Medical Sciences, Institute of Health Policy and Management, Erasmus University Rotterdam, P.O. Box 1738, Rotterdam, DR 3000, The Netherlands; 2 Argos Zorggroep, P.O. Box 4023, Schiedam, GA 3102, The Netherlands; 3 Department of Public Health, Erasmus Medical Center Rotterdam, P.O. Box 2040, Rotterdam, CA 3000, The Netherlands

## Abstract

**Background:**

It is known that interprofessional collaboration is crucial for integrated care delivery, yet we are still unclear about the underlying mechanisms explaining effectiveness of integrated care delivery to older patients. In addition, we lack research comparing integrated care delivery between hospitals. Therefore, this study aims to (i) provide insight into the underlying components ‘relational coordination’ and ‘situational awareness’ of integrated care delivery and the role of team and organizational context in integrated care delivery; and (ii) compare situational awareness, relational coordination, and integrated care delivery of different hospitals in the Netherlands.

**Methods:**

This cross-sectional study took place in 2012 among professionals from three different hospitals involved in the delivery of care to older patients. A total of 215 professionals filled in the questionnaire (42% response rate).Descriptive statistics and paired-sample t-tests were used to investigate the level of situational awareness, relational coordination, and integrated care delivery in the three different hospitals. Correlation and multilevel analyses were used to investigate the relationship between background characteristics, team context, organizational context, situational awareness, relational coordination and integrated care delivery.

**Results:**

No differences in background characteristics, team context, organizational context, situational awareness, relational coordination and integrated care delivery were found among the three hospitals. Correlational analysis revealed that situational awareness (r = 0.30; p < 0.01), relational coordination (r = 0.17; p < 0.05), team climate (r = 0.29; p < 0.01), formal internal communication (r = 0.46; p < 0.01), and informal internal communication (r = 0.36; p < 0.01) were positively associated with integrated care delivery. Stepwise multilevel analyses showed that formal internal communication (p < 0.001) and situational awareness (p < 0.01) were associated with integrated care delivery. Team climate was not significantly associated with integrated care delivery when situational awareness and relational coordination were included in the equation. Thus situational awareness acted as mediator between team climate and integrated care delivery among professionals delivering care to older hospitalized patients.

**Conclusions:**

The results of this study show the importance of formal internal communication and situational awareness for quality of care delivery to hospitalized older patients.

## Background

Currently, health care delivery in hospitals often leads to poor outcomes for older patients [1]. Many hospitalized older patients suffer from a mixture of problems and therefore are expected to benefit from integrated care delivery. This holistic and personalized care encompasses the total care process, rather than focusing on disease-related problems only [2-5]. The patient should be placed in the centre of the care process and care should be tailored to their personal needs. Interprofessional collaboration among professionals from a variety of disciplines is considered to be critical in integrated care delivery due to the many interdependent work requirements [6,7]. To provide care that is holistic and patient-centered responding to the multidimensional health needs of older patients more is needed than professionals who each work within their particular scope of practice and interact formally (multidisciplinary teamwork), but rather professionals who have some overlapping of professional roles, communicate and coordinate together in their care of older patients and share problem solving and decision making (interprofessional collaboration) [8,9]. In this way, the coordinated response of all activities and information to the needs of older patients is organized through horizontal work processes, rather than through functional profiles. Besides medical expertise, interprofessional collaboration is crucial for integrated care delivery [10-14]. Yet we are still unclear about the underlying mechanisms that explain how integrated care enhances the quality of care delivery to older patients.

### Conceptual model: underlying mechanisms of integrated care delivery

**Figure 1:**
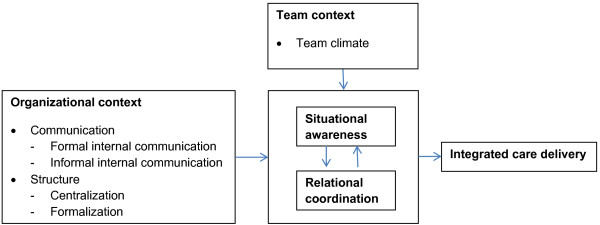
Underlying mechanisms of integrated care delivery in hospitals.

Figure 1 displays our conceptual model with the underlying components ‘relational coordination’ and ‘situational awareness’ of integrated care delivery. We expect that the organizational context as well as team context influence relational coordination, situational awareness and integrated care delivery.

#### Team context

Interprofessional collaboration and multidisciplinary teamwork are expected to benefit from a positive team climate. With a supportive climate for teamwork, team members are more willing to share resources, perceptions, policies, practices, and procedures [15]. As such, a team climate may encourage social interaction and draws the interpretations by professionals of events and objects closer together [16,17]. Consequently, professionals working in such teams may coordinate and communicate more freely with each other regarding their tasks and expertise [18]. An encouraging team climate is therefore expected to enhance integrated care delivery.

#### Organizational context

The structure of an organizations’ internal communication channels may have consequences for the exchange and transfer of knowledge. Internal communication has two components: on one side, *formal internal communication* which consists of formal activities for teams and units [19], and on the other side *informal internal communication* which consists of a more casual form of information sharing typically used in personal conversations [20]. To enhance integrated care delivery, internal communication should be channelled in such a way that professionals have access to diverse sources of new information and knowledge through e.g. frequent multidisciplinary team meetings and electronic information systems [21].

The amount of *centralization* and *formalization* within an organization may also affect quality of integrated care delivery. Research has shown that less formal, functionally differentiated organizations with decentralized decision making and a great variety of professionals are more likely to generate, and develop new knowledge [22]. We therefore reason that these organizations create the possibility for professionals to combine the knowledge needed for integrated care delivery by shared problem solving and decision making.

#### Relational coordination

Since integrated care delivery is characterized by overlapping care processes performed by multiple professionals, the complementary role of each health care professional and the interdependency among them are important features [23,24]. According to the theory of *relational coordination*, the effectiveness of coordination is determined by the quality of communication among professionals in a work process, which depends on the quality of their underlying relationships [25]. The quality of their relationships, in turn, reinforces the quality of their communication. Relational coordination is defined as ‘a mutually reinforcing process of interaction between communication and relationships carried out for the purpose of task integration’ [26]. More simply, relational coordination is coordinating work through relationships of shared goals, shared knowledge and mutual respect, supported by frequent, timely, accurate, and problem solving communication [25]. Together, these communication and relational dynamics provide the basis for coordinated collective action under conditions of task interdependence, uncertainty, and time constraints [27]. Since the provision of care to elderly is a complex undertaking that requires input from and high levels of interdependency among professionals from various disciplines [28], it can be reasoned that relational coordination improves integrated care delivery by enhancing the exchange of relevant information and by strengthening shared goals. Research indeed showed that relational coordination was associated with better quality of care in the primary care setting [12,29], community setting [12], and hospital setting [30,31].

#### Situational awareness

For coordination to operate effectively and improve quality of care delivery, it is important that professionals are aware of the individual patients’ demand for care. *Situational awareness* is the level of awareness that an individual has of a situation; a dynamic understanding of “what’s going on”. As part of information processing, situational awareness follows perception of the situation and leads to decision making and action execution [32]. This dynamic knowledge is especially important in the health care context where misinformation can result in negative consequences for the patient. Individual treatment plans are made by integrating information, from a variety of sources such as assessment of the patient, information from charts and monitors, and other professionals with individual knowledge. The professionals then comprehend the meaning and significance of the patient assessment and project this onto likely outcomes. These expectations result from awareness of the situation of the patient and play a critical role in the integrated decision making process of the individual patient, in terms of actions to be taken or in some cases not taken [33-35]. As such they are expected to enhance integrated care delivery.

### Aims

The underlying mechanisms explaining high quality integrated care delivery to vulnerable elderly are still unknown. In addition, we lack research comparing integrated care delivery between hospitals. Therefore, this study aims to (i) provide insight into the underlying components ‘relational coordination’ and ‘situational awareness’ of integrated care delivery and the role of team and organizational context in delivering integrated care; and (ii) compare situational awareness, relational coordination, and integrated care delivery among three hospitals in the Netherlands.

## Methods

### Setting and design

This cross-sectional study was performed as part of a larger evaluation study examining the delivery of integrated care to hospitalized older patients in The Netherlands. Data were collected in 2012 by means of questionnaires distributed in three different hospitals. Since no clear distinction could be made between professional teams within the hospital delivering care to older patients, data were collected on a unit level. Professionals involved in the delivery of care to older patients were invited to complete the questionnaire (215 out of 510 respondents, overall response rate 42%). These professionals received a questionnaire by mail and a few weeks later a reminder and questionnaire was send to non-responders with a gift voucher of 10 euro as incentive for participation. The response and non-response of respondents were evenly distributed throughout the hospitals and hospital units. Written informed consent was obtained from the professionals for the publication of this report and any accompanying images.

Table 1 describes the three different settings. Hospital A implemented the Prevention and Reactivation Care Program (PReCaP) in three units (geriatrics, cardiology, and internal medicine). This program was designed to prevent loss of function in older patients due to hospitalization and targeted older hospital patients (≥ 65 years of age) who were vulnerable to loss of function after hospital admission [36]. The program utilized a multidisciplinary, integrated, and goal-orientated approach focused at the early screening of risk factors for functional decline and the provision of a patient-orientated reactivation program [37]. Hospital B, that participated with the units of internal medicine, respiratory medicine, neurology, orthopedics and general surgery, did not implement the integrated care program. Neither did hospital C, that participated with the units of internal medicine, respiratory medicine, neurology, orthopedics and cardiology. Work processes focused on the patients’ medical condition and each involved medical discipline used a separate treatment plan, without active integration. However, Hospital B did use an electronic patient record including targeted consultation and central intake prior to admission. A clinical geriatrician was available in Hospital A and B, but not in Hospital C.

**Table 1:** Differences between the prevention and reactivation care program and two other hospitals in The Netherlands

	**Hospital A - prevention and reactivation care program**	**Hospital B**	**Hospital C**
Hospital care	Identification of vulnerable older patient within 48 h	Start reactivation treatment after discharge	Start reactivation path after discharge
	Assessment of risk factors for functional decline		
	Start reactivation treatment within 48 h	Medication safety project	Medication safety project
	Clinical geriatrician	Clinical geriatrician (consultation two days a week)	
	Geriatric nurses		
		Electronic patient record including targeted consultation (consult dietitian based on SNAQ scores)	
		Central intake prior to admission including screening frail elderly and development individualized care plan	
Multidisciplinary approach	Weekly multidisciplinary team meeting	Key professional is responsible for treatment and interdisciplinary consults	Key professional is responsible for treatment and interdisciplinary consults
	Treatment and care focused on medical condition		
	and functioning in six domains (i.e. physical, mental, social, financial, home, and care)		
		Discussion and coordination focused on medical condition	Discussion and coordination focused on medical condition
	Goal-orientated approach		
Patient	Patient orientated integrated treatment plan	Separate treatment plans	Separate treatment plans
	Discussion treatment with patient during entire treatment path	Treatment coherence determined by patient	Treatment coherence determined by patient
	Problem solving		

It was expected that hospital A would score higher on overall integrated care delivery in comparison to hospital B and C. Situational awareness was expected to be increased, due to the patient-orientated integrated treatment plan and by discussing the treatment with the older patient. Weekly multidisciplinary team meetings and the goal-orientated approach are expected to result in higher levels of relational coordination between professionals who deliver care to the older patient.

### Questionnaires

#### Integrated care delivery

The Assessment of Chronic Illness Care Short version (ACIC-S, see Additional file 1: Table S1) was originally developed to measure the degree to which a healthcare system adheres to all six elements of the Chronic Care Model (CCM), and the integration effect that occurs when all model elements are engaged [38]. The ACIC-S is responsive to the system changes made by teams [12]. Four subscales of the six subscales were used in the current study, addressing self-management support (3 items), delivery system design (3 items), decision support (3 items), and clinical information systems (3 items) [38]. Since we investigated integrated care delivery to hospitalized older patients the subscales community and health systems where less suitable/applicable. Since chronic illness care is a complex undertaking that contains several interacting components, partly performed within the hospital [39], we generalized the ACIC-S to the current setting of integrated care delivery for hospitalized older patients. Responses were structured on a scale of 0–11, with higher scores indicating more comprehensive integrated care delivery. ACIC-S scores indicate: 0–2 (little or no support for integrated care), 3–5 (basic or intermediate support for integrated care), 6–8 (advanced support for integrated care), and 9–11 (optimal or comprehensive integrated care). Cronbach’s alpha for the overall ACIC-S in this study was 0.90.

#### Situational awareness

The Situation Awareness Global Assessment Technique (SAGAT, see Additional file 1: Table S2) is based on a three-level model of situational awareness. It addresses perception of the elements (3 items), comprehension of their meaning (3 items), and projection of future status (3 items) [40]. Although developed specifically to assess pilot situational awareness [41,42], the SAGAT has been used in the hospital setting to e.g. measure nurses’ ability to assess and manage patient deterioration, and the integration of patient information [43,44]. Responses were structured on a scale of 0–5, with higher scores indicating more situational awareness. The overall Cronbach’s alpha was 0.92.

#### Relational coordination

Relational coordination was measured using six survey questions on a four-point scale (1 = never, 2 = rarely 3 = occasionally, and 4 = all the time) including three questions about communication (frequency/timeliness, accuracy, problem-solving) and three questions about relationships (shared goals, shared knowledge, mutual respect, see Additional file 1: Table S3). The relational coordination score was derived by calculating the mean of the item scores. Higher scores indicated better or more desirable relational coordination [12,29,30,45]. The questionnaire was originally developed to measure airline operation [46], and has been applied in hospitals [27]. Pilot testing revealed that the items ‘timely’ and ‘frequent’ communication were not distinguishable for the professionals delivering care to hospitalized older patients, which led us to combine both aspects of relational coordination in a single question. In the current study, respondents were asked about communication and coordination with other professionals involved in delivering care to hospitalized older patients: medical specialists, nurses, physical therapists, dieticians, social workers, transfer nurses, case managers, and family physicians. Cronbach’s alpha for the adjusted questionnaire used in this study was 0.94.

#### Team context

A short version of the Team Climate Inventory (TCI) was used to measure the professionals’ perceptions of team climate while working in multidisciplinary teams delivering care to older patients. The questionnaire comprises four broad factors reflecting a team’s shared perceptions of organizational policies, practices and procedures: shared vision and objectives (4 items), participative safety (4 items), task orientation (3 items) and support for innovation (3 items). Participants were asked to rate their agreement on the TCI-items on a 5-point scale ranging from 1 (strongly disagree) to 5 (strongly agree). Higher scores indicated a better or more desirable team climate [47-49]. The overall Cronbach’s alpha for the short version of the TCI in this study was 0.89.

#### Organizational context

In order to provide insight into the organizational context, questions were asked about communication and the structure of decision making with the organization. All questions were rated on a 7-point scale ranging from 1 (strongly disagree) to 7 (strongly agree).

Communication was measured by asking participants about the communication channels within their organization [50,51]. Subscales were formal internal exchange of information (6 items), and informal internal exchange of information (3 items, see Additional file 1: Table S4). Examples were “Normally, meetings are held to share knowledge, to share ideas, and discuss issues related to work”, and “In our organization, there is ample opportunity for informal hall talk”. Cronbach’s alpha was 0.77 for formal internal exchange of information. And Cronbach’s alpha was 0.84 for informal internal exchange of information.

The organizational structure was measured by the amount of centralization using three items [52]. An example was “Little action can be taken until a supervisor approves a decision”. Cronbach’s alpha was 0.60. Formalization was measured with three items. “How things are done here is left up to the persons doing the work” was an example. Cronbach’s alpha was 0.62.

#### Background characteristics

In addition, we asked participants for gender, occupational background, and the number of years they worked in their organization.

### Data analysis

Descriptive statistics were used to analyze professionals’ background characteristics, the team and organizational context, and the level of integrated care delivery, relational coordination and situational awareness. We tested the levels of situational awareness, relational coordination, and integrated care delivery in the three different hospitals. The degree to which differences existed was assessed through a series of paired-sample t-tests. Correlation analysis was used to investigate the relationship between the background characteristics, team and organizational context, situational awareness, relational coordination, and integrated care delivery. We tested for influence of unit (level 2) on integrated care delivery. These results indicated that unit affects integrated care delivery (-2 loglikelihood 754.456 *vs.* 743.369: *p* = 0.01). Therefore, to account for the hierarchical structure of the study design we fitted a hierarchical random-effects model. The hierarchical structure comprises of 215 professionals nested in 13 teams. Individuals were excluded if any outcome observation was missing, leading to a total of 189 professionals in the multilevel regression analysis. To assess the extent to which variance should be ascribed to the unit rather than to the individual, unit was added in model 1. We introduced the team and organizational context in model 2 and situational awareness and relational coordination in model 3. In addition, team climate, relational coordination and situational awareness were aggregated on unit level and added to the analysis. This did not have a significant influence on the results. Deviance tests or likelihood ratio tests were used to compare the relative fit of the different models. A significance level of 0.05 was used for all statistical tests. Data were analyzed using the SPSS software package (ver. 18.0 for Windows; SPSS Inc., Chicago, IL, USA).

### Ethics approval

The study protocol was approved by the Medical Ethics Committee of the Erasmus Medical Centre, Rotterdam, the Netherlands, under protocol number MEC 2011–041.

## Results

The eligible study population consisted of 510 professionals, 215 of whom completed the questionnaire (42% response rate). The respondents were distributed in three hospitals, with a response rate of 41% (52 out of 128) in hospital A, 44% (121 out of 274) in hospital B, and 39% (42 out of 108) in hospital C. Of those who completed the questionnaire, the majority of respondents in all hospitals was female (between 76.2% and 90.0%), and worked as a nurse (between 71.7% and 84.6%). Table 2 displays the descriptive characteristics (mean and standard deviation) of the total study population and per hospital. The overall mean score for integrated care delivery on a 0–11 scale was 5.44 (± 1.79), indicating that basic support for integrated care delivery was present. On a 0–5 scale, the overall mean score for situational awareness was 3.91 (± 0.61). On a 1–4 scale, the overall mean score for relational coordination was 2.97 (± 0.60).

**Table 2:** Descriptive statistics

**Characteristics**	**Range**	**Overall (*n*** **= 215) % or mean (SD)**	**Hospital A (*n*** **= 52) % or mean (SD)**	**Hospital B (*n*** **= 121) % or mean (SD)**	**Hospital C (*n*** **= 42) % or mean (SD)**
Gender (female)		86.3%	90.0%	88.3%	76.2%
Profession					
Medical specialist		7.5%	10.0%	6.6%	7.7%
Nurse		77.5%	79.2%	71.1%	84.6%
Paramedic		15.0%	10.8%	22.3%	7.7%
Years working in the organization (> 5 years)		59.2%	46.2%	74.8%	70.7%
Integrated care delivery (ACIC-S)^a^	0-11	5.44 (1.79)	5.53 (1.94)	5.48 (1.72)	5.21 (1.81)
Situational awareness	1-5	3.91 (0.61)	3.98 (0.61)	3.89 (0.58)	3.88 (0.72)
Relational coordination	1-4	2.97 (0.60)	3.12 (0.64)	2.93 (0.57)	2.91 (0.63)
*Team context*					
Team climate	1-5	3.53 (0.58)	3.54 (0.53)	3.47 (0.57)	3.68 (0.63)
*Organizational context*					
Communication					
Formal internal communication	1-7	4.14 (1.00)	4.12 (1.11)	4.24 (0.95)	3.80 (0.96)
Informal internal communication	1-7	4.95 (1.22)	4.71 (1.43)	5.15 (1.14)	4.69 (1.08)
Structure					
Centralization	1-7	3.25 (1.18)	3.11 (1.21)	3.23 (1.18)	3.45 (1.13)
Formalization	1-7	4.02 (1.07)	4.21 (1.10)	3.85 (1.03)	4.23 (1.09)

*Note.* SD = standard deviation. ACIC-S; Assessment of Chronic Illness Care Short Version. ^a^ACIC-S scores indicate: 0–2 (little or no support for integrated care), 3–5 (basic or intermediate support for integrated care), 6–8 (advanced support for integrated care), and 9–11 (optimal or comprehensive integrated care).

### Comparison between hospitals

The three hospitals did not differ significantly with regard to the instruments used in this study (all *p* > 0.05) (Table 2). The different hospital units did however differ on integrated care delivery (*p* < 0.001), with geriatrics in hospital A (mean 6.80; ± 1.40), respiratory medicine in hospital B (mean 6.22; ± 1.49), neurology in hospital C (mean 6.43; ± 1.47) and orthopedics in hospital B and C (mean 6.33; ± 1.28 and mean 6.18; ± 0.58) scoring significantly higher than the other hospital units (overall mean 5.44; ± 1.70). The hospital units did also differ on informal internal communication (*p* < 0.05), with respiratory medicine in hospital B (mean 5.23; ± 1.14), neurology in hospital B (mean 5.52; ± 1.14) and orthopedics in hospital B and C (mean 5.57; ± 0.90 and mean 5.40; ± 0.44) scoring significantly higher than the other hospital units (overall mean 4.95; ± 1.22).

### Associations with integrated care delivery

Correlation analysis revealed that situational awareness (*r* = 0.30; *p* < 0.01), relational coordination (*r* = 0.17; *p* < 0.05), team climate (*r* = 0.29; *p* < 0.01), formal internal communication (*r* = 0.46; *p* < 0.01), and informal internal communication (*r* = 0.36; *p* < 0.01) were positively associated with integrated care delivery (Table 3).

**Table 3:** Associations with integrated care delivery

	**Integrated care delivery**	***n***
Gender (female)	-0.02	186
Medical specialists	-0.06	177
Nurse	-0.06	177
Paramedic	0.12	177
Years working in the organization (> 1 year)	-0.11	189
Situational awareness	0.30**	194
Relational coordination	0.17*	188
*Team context*		
Team climate	0.29**	170
*Organizational context*		
Communication		
Formal internal communication	0.46**	181
Informal internal communication	0.36**	186
Structure		
Centralization	0.01	176
Formalization	-0.13	179

*Note.* **p < 0.01; *p < 0.05 (two-tailed).

The results of the stepwise multilevel analyses are displayed in Table 4. The first (empty) model served as a baseline with just intercepts. Model 2 showed that team climate (*p* < 0.01) and formal internal communication (*p* < 0.001) had a positive effect on integrated care delivery. When situational awareness and relational coordination were added to model 3, the results showed that in addition to formal internal communication (*p* < 0.001), situational awareness (*p* < 0.01) predicted integrated care delivery. Team climate was not significantly associated with integrated care delivery when situational awareness and relational coordination were included in the equation. Thus situational awareness acted as mediator between team climate and integrated care delivery among professionals delivering care to older hospitalized patients.

**Table 4:** Hierarchical multilevel analyses of factors associated with integrated care (random intercepts model) (n = 189)

**Model**	**1**				**2**				**3**			
	**B**	**SD**	**ß**	**SE**	**B**	**SD**	**ß**	**SE**	**B**	**SD**	**ß**	**SE**
Constant	5.49	0.21	0.03	0.12	0.59	0.82	0.05	0.09	-0.93	1.04	0.07	0.09
*Team context*												
Team climate					0.48*	0.22	0.16*	0.07	0.21	0.24	0.07	0.08
*Organizational context*												
Formal internal communication					0.60**	0.15	0.34**	0.08	0.59**	0.14	0.33**	0.08
Informal internal communication					0.15	0.12	0.10	0.08	0.13	0.11	0.09	0.08
Situational awareness									0.71*	0.23	0.24*	0.08
Relational coordination									-0.03	0.24	-0.01	0.08
-2 log likelihood	743.369				598.619				589.42			

^*^p ≤ 0.01; **p ≤ 0.001 (two-tailed). SD = Standard Deviation; SE = standard error. Listwise deletion of missing cases resulted in 189 cases for the multilevel regression analyses.

## Discussion and conclusion

In this study, we aimed to provide insight into the underlying components ‘relational coordination’ and ‘situational awareness’ of integrated care delivery and the role of team and organizational context in delivering integrated care. We found that awareness of the individual situation of patients was associated with higher levels of integrated care delivery. A greater understanding of patients’ personal needs and the roles of various disciplines to fulfill these needs may have resulted in a more coordinated and integrated response by the involved professionals. Situational awareness has the objective of understanding professional focus and intentions. Perception of the actual situation of the patient (e.g. awareness of the current health condition), in combination with a comprehension of what might be necessary for the patient (e.g. knowledge about different treatment options), and a projection of what might happen (e.g. how to react to sudden deterioration) make it possible for professionals to react to individual patient needs [33-35], which is expected to lead to better integrated care delivery.

Organization of formal activities that emphasize internal communication between professionals with different occupational backgrounds are also associated with higher levels of integrated care delivery in this study. Knowledge sharing is known to be one of the key mechanisms by which internal communication takes place [53]. Professionals who are provided with the opportunity to connect with other professionals through formal activities may expand their professional knowledge and skills [54]. Formal arrangement of face-to-face discussion may be an important way for hospitals to stimulate professionals to share new ideas and insights and keep professionals up-to-date about developments [55]. As such these formal arrangements of communication between professionals from different occupational backgrounds are expected to improve integrated care delivery [55,56].

While we did not find a significant relationship between relational coordination and integrated care delivery in the multivariate analyses the univariate analyses did reveal a significant relationship. Team climate and situational awareness might have mediated the relationship between relational coordination and integrated care delivery.

The three Dutch hospitals did not differ in the degree of integrated care delivery, all scoring basic or intermediate support for integrated care. To understand the outcome, the work processes in the different hospitals were compared. Hospital A, which implemented the integrated care program, introduced weekly multidisciplinary team meetings. These meetings made it possible for professionals to share information about the patients’ situation and demand for care, after which a patient-orientated integrated treatment plan was made. While hospital B and C did not implement these multidisciplinary team meetings; professionals in charge of care delivery did seek information from other professionals through interdisciplinary consults. Maybe introducing multidisciplinary team meetings had the same effects on integrated care delivery as the use of interdisciplinary consults. In line with this, the professionals in all hospitals worked according to treatment plans. The treatment plans in hospital A were patient-orientated and diverse disciplines were integrated. Hospital B and C worked with separate treatment plans for each discipline. But since the professionals in hospital B and C actively sought information from others, one could question whether their treatment plans were indeed less integrated than the treatment plans of hospital A. In contrary to hospital A, hospital B and C did not implement a screening instrument for vulnerability of older patients. Yet, they did perform a basic screening for general health problems and took proactive measures when problems were suspected. One could reason that both screening instruments had the same effect on the choice of treatment for the intervention and control hospitals, and therefore no differences in the care processes were identified. In addition, hospital B and C are participating in quality improvement programs other than serving as a control group in the current study (e.g. improving patient safety by medication verification). The participation in these projects could alter the perception of professionals on the quality of care they deliver. This could be of influence on their responses to the questionnaires of the current study. It should also be noted that hospital B is a Dutch training hospital for medical residents. Nowadays, the necessity of coordination for health care delivery is emphasized during training [57], which could have resulted in higher levels of care integration. In addition, hospital C is smaller compared to the other two hospitals. Research has shown that smaller hospitals show higher levels of cooperation [58]. Working with fewer professionals creates less boundaries for information sharing and decision making, which may have resulted in higher levels of integrated care delivery. However, less formalized communication may also have negative effects for some units or for some patients [59].

While no differences were found between the hospitals, the hospital units did differ in their level of integrated care delivery. Elsewhere we reported higher levels of integrated care delivery in a geriatric hospital unit suggesting that professionals are more used to integrated care delivery (e.g. by participating in multidisciplinary team meetings), than professionals in other units [31]. One should therefore take differences in integrated care delivery between units into account when analyzing what is needed for integrated care delivery in the hospital setting.

The limitations of this study should be considered when interpreting the findings. Firstly, the cross-sectional design allowed us to identify associations but not to determine causality. Longitudinal data would provide the opportunity to disentangle the dynamic relationships among situational awareness, relational coordination and integrated care delivery. Secondly, the response rate of 42% may have led to potential non-response bias. However, it is only slightly below the average response rate of about 50%, which is often found among professionals working in hospitals [60]. Thirdly, we were not able to control for all contextual factors that may be of importance for integrated care delivery. Earlier research has shown that e.g. unit size, availability of support services, work complexity and work engagement have an influence on care delivery [61,62]. Fourthly, the management of hospital A allowed only three of the ten hospital units in that hospital to participate in the integrated care program and current study. It might have been possible to detect a stronger effect of the program when it would have been implemented throughout the whole hospital. And finally, although we examined the relationship between situational awareness, relational coordination and integrated care delivery, the link between situational awareness and relational coordination remains unclear. Earlier research has shown that relational coordination improves the exchange of information relevant for delivering high-quality care [30]. Furthermore, Endsley [63-65] argues that situational awareness serves as an index for coordination or interface effectiveness. Future research has to further explore this dynamic relationship between situational awareness and relational coordination. In addition, further research is necessary to assess the effects of integrated care delivery on improved patient experiences and outcomes.

We can conclude that the current study provides insight into the underlying mechanisms of integrated care delivery in hospitals. Awareness of the individual patients’ situation and structured activities within the hospital that enhance information sharing are a necessity for placing the older patient in the center of the care process. To enhance integrated care delivery hospitals should therefore create formal moments of communication among professionals of different occupational backgrounds. In addition, training programs should especially devote time to teaching individual skills related to situational awareness.

## Competing interests

The authors declared that they have no competing interest.

## Authors’ contributions

All authors: 1) made substantial contributions to conception and design, or acquisition of data, or analysis and interpretation of data; 2) were involved in drafting the manuscript or revising it critically for important intellectual content; and 3) gave final approval for the version to be submitted.

## Pre-publication history

The pre-publication history for this paper can be accessed here:

http://www.biomedcentral.com/1471-2318/14/3/prepub

## Supplementary Material

Additional file 1 Survey questions. Click here for file
