# Supplementary material for: Situational awareness, relational coordination and integrated care delivery to hospitalized elderly in The Netherlands: a comparison between hospitals
Source: BMC Geriatr. 2014 Jan 10;14:3. doi: 10.1186/1471-2318-14-3 (PMC3890569; doi:10.1186/1471-2318-14-3)
Supplement: Additional file 1 — Survey questions. [file 1471-2318-14-3-S1.docx]

**Additional file 1: Table S1 ACIC-S scale**

| *Self-management support* | |
| --- | --- |
| 1. | Assessment and documentation of self-management needs and activities |
| 2. | Self-management support |
| 3. | Addressing concerns of patients and families |
| *Decision support* | |
| 4. | Evidence-based guidelines |
| 5. | Providing education for elderly care |
| 6. | Informing patients about guidelines |
| *Delivery system design* | |
| 7. | Appointment system |
| 8. | Planned visits for elderly care |
| 9. | Continuity of care |
| *Clinical information systems* | |
| 10. | Feedback |
| 11. | Information about relevant subgroups of patients needing services |
| 12. | Patient treatment plans |

**Additional file 1: Table S2 SAGAT scale**

| *Perception of the elements* | |
| --- | --- |
| 1. | Are other professionals aware of the current health condition of the elderly patient? |
| 2. | Do other professionals know which medicine the elderly patient takes? |
| 3. | Do other professionals know which treatment the elderly patient undergoes? |
| *Comprehension of their meaning* | |
| 4. | Do other professionals have sufficient information about the general health condition of the elderly patient? |
| 5. | Do other professionals know which treatment options the elderly patient has? |
| 6. | Do other professionals know which treatment goals the elderly patient has? |
| *Projection of future status* | |
| 7. | Do other professionals have expectations about the course of illness of the elderly patient? |
| 8. | Do other professionals know how to act when sudden deterioration occurs in the situation of the elderly patient? |
| 9. | Do other professionals how to contact when sudden deterioration occurs in the situation of the elderly patient? |

**Additional file 1: Table S3 Relational Coordination survey**

| 1. | How frequently/timely do people in each of these groups communicate with you about the care for elderly patients? |
| --- | --- |
| 2. | Do people in these groups communicate with you accurately about the care for elderly patients? |
| 3. | When there is a problem with elderly patients, do people in these groups blame others or work with you to solve the problem? |
| 4. | Do people in these groups share your goals for the care for elderly patients? |
| 5. | Do people in these groups know about the work you do with elderly patients? |
| 6. | Do people in these groups respect the work you do with elderly patients? |

**Additional file 1: Table S4 Internal communication**

| *Formal internal communication* | |
| --- | --- |
| 1. | Staff rotation programs exist between the organization’s different functional areas. |
| 2. | Normally, meetings are held to share knowledge, to share ideas, and discuss issues related to work. |
| 3. | The organization’s files and databases provide the necessary information for carrying out work. |
| 4. | Procedures exist within the firm for gathering, adding and internally distributing proposals from employees. |
| 5. | Suggestions made by patients are often incorporated into processes of care delivery. |
| 6. | The organization periodically brings out written progress reports, distributed among all the staff. |
| *Informal internal communication* | |
| 7. | In our organization, there is ample opportunity for informal hall talk. |
| 8. | In this unit, employees from different departments feel comfortable calling each other when the need arises. |
| 9. | In this organizational unit, it is easy to talk with virtually  anyone you need to, regardless of rank or position. |
